# High*-*yield hybrid breeding of *Camellia oleifolia* based on ISSR molecular markers

**DOI:** 10.1186/s12870-024-05218-x

**Published:** 2024-06-08

**Authors:** Jinjia Zheng, Haiqi Su, Shaosheng Pu, Hui Chen, Yousry A. El-Kassaby, Zhijian Yang, Jinling Feng

**Affiliations:** 1 College of Forestry, Fujian Agriculture and Forestry University, Fuzhou, 350002 China; 2 Department of Forest and Conservation Sciences, Faculty of Forestry, University of British Columbia, 2424 Main Mall, Vancouver, BC V6T 1Z4 Canada

**Keywords:** *Camellia oleifera*, Molecular marker assisted-breeding, Marker-association analysis, ISSR marker development, High-yield early identification

## Abstract

**Background:**

*C. Oleifera* is among the world’s largest four woody plants known for their edible oil production, yet the contribution rate of improved varieties is less than 20%. The species traditional breeding is lengthy cycle (20–30 years), occupation of land resources, high labor cost, and low accuracy and efficiency, which can be enhanced by molecular marker-assisted selection. However, the lack of high-quality molecular markers hinders the species genetic analysis and molecular breeding.

**Results:**

Through quantitative traits characterization, genetic diversity assessment, and association studies, we generated a selection population with wide genetic diversity, and identified five excellent high-yield parental combinations associated with four reliable high-yield ISSR markers. Early selection criteria were determined based on kernel fresh weight and cultivated 1-year seedling height, aided by the identification of these 4 ISSR markers. Specific assignment of selected individuals as paternal and maternal parents was made to capitalize on their unique attributes.

**Conclusions:**

Our results indicated that molecular markers-assisted breeding can effectively shorten, enhance selection accuracy and efficiency and facilitate the development of a new breeding system for *C. oleifera*.

**Supplementary Information:**

The online version contains supplementary material available at 10.1186/s12870-024-05218-x.

## Introduction

Woody plants traditional breeding is a long-term endeavor characterized by lengthy cycles and low efficiency [1]. The genetic control of complex quantitative traits (e.g., growth and fruit yield) along with their interaction with environmental factors, further complicates traditional breeding, resulting is slower incremental gain over generations [2, 3]. The development of molecular marker-assisted selection (MAS) is expected to enhance breeding effectiveness through shortening breeding cycles [3, 4]. Through linkage disequilibrium, association analysis directly identifies specific marker sites closely associated with phenotypic variation, thereby linking the diversity of target traits with genes’ polymorphisms [5]. Association analysis, characterized by its efficiency, wide application and high precision, is among the main methods to analyzing genotypes of plant quantitative traits, and is widely used in important tree species such as *Prunus persica, Populus, Pterocarya stenoptera, Vitis berlandieri* [5–8].

*Camellia oleifolia* is one of the four major woody edible oil species in the world [9]. By 2021, in China alone, camellia planting area has reached 4.592 × 10^6^ hm^2^, yielding 3.94 million tons seed production annually, resulting in an annual oil output of 889,000 tons with industry output exceeding 27 billion US dollars [10]. Despite these figures, China still relies heavily on importing edible oil to meet local demand. Camellia oil plays an important role in augmenting the edible oil supply and aiding rural revitalization efforts [10, 11]. *C. oleifolia* conventional breeding has made great progress, but the contribution rate of improved varieties is less than 20% [12]. Thus, there is a pressing need to accelerate *C. oleifolia* traditional breeding for high yield. Most yield phenotypic traits can only be assessed at the flowering and bearing stage of hybrid plants, which can lead to long breeding cycle, occupation of land resources, high labor cost, and low accuracy and efficiency selection, which can be solved by MAS [3, 13, 14]. Molecular markers are neutral and exist in different tissues and development stages, irrespective of environmental conditions [15]. So, the development molecular markers applications are particularly important in MAS breeding of *C. oleifolia.*

ISSR (Inter Simper Sequence Repeat) is based on SSR, involving the addition of nucleotides at the sequence end, amplification of the repeated DNA sequence, and acquisition of a dominant amplified band. Polymorphism is determined based on the specificity of the amplified fragment size [15, 16]. Compared with other molecular markers, ISSR have the characteristics of generating large amount of information, high polymorphism, efficient and stable results, easy operation, fast and lower cost [17, 18]. ISSR have been widely used in assessing plant genetic diversity [19, 20], genetic relationship [16], variety identification [21] and association analysis [18, 22]. *C. oleifolia* is a highly heterozygous polyploid plant with complex genetic basis, wide distribution, and large regional differences [23]. The lack of high-quality molecular markers hinders the genetic analysis and molecular breeding of *C. oleifolia* important traits, and brings great difficulties and challenges to the species MAS breeding [14, 24, 25]. Fruit yield is one of the most important quantitative traits of *C. oleifolia* as it involves interaction of multiple genes [23, 26]. There are no reports on the development of molecular markers closely linked with yield traits for *C. oleifolia* [14, 27–30].

**Fig. 1:**
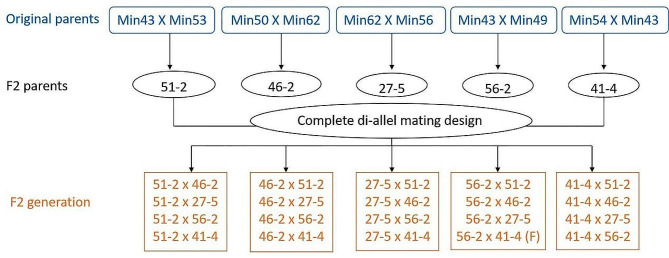
The schematic of *C. oleifera* crossbreeding. F: the failure of pollen hybridization

Here, we constructed 570 *C. oleifolia* F2 generation population of 19 hybrid combinations each with 30 plants originated from 5 parental individuals as selection materials (see Fig. 1, for complete pedigree development) over 50 years. We analyzed the variation of 28 traits at the seedling and full fruit stages, assessed the developed ISSR markers genetic diversity of the 19 hybrid combinations, and identified high-yield combinations. Through association analysis, combining with correlation analysis of yield per plant and other traits, excellent high-yield and tightly interlocked ISSR molecular markers were selected. Through the identification of excellent high-yield markers in the F2 generation early selection method was determined, and a molecular-assisted breeding system was designed to achieve the improvement needed of *C. oleifolia* improved breeding method.

## Results

### Variation and correlation of phenotypic traits in the 19 F2 families at the seedling stage

The studied eight phenotypic traits (H_S_, D_S_, H_S_/D_S,_ Tr, Gs, Pn, Ci, and WUE) of the 19 F2 families produced CV range of 10.63 ∼ 37.95%, notably all greater than 10% (Table S1). The CV values of seedling morphological indices (H_S_, D_S_, and H_S_/D_S_) ranged between 10.63 and 17.85%, with H_S_/D_S_ having the largest value. Additionally, the CV values of photosynthetic physiological indices ranged between 26.99 and 37.95%, with WUE showing the largest value. These high CV values indicate that the F2 generation harbored abundant seedling phenotypic traits variation. The skewness and kurtosis of seedling traits’ absolute values were less than 3, indicating that these traits produced continuous normal distribution.

The correlation between morphological (H_S_, D_S_, and H_S_/D_S_) and photosynthetic (Tr, Gs, Pn, Ci, and WUE) traits of the 19 F2 families indicated the presence of significant positive correlation between H_S_ and Pn and Ci, as well as significant negative correlation with WUE (Table 1). Additionally, D_S_ correlated positively with Tr and Gs. Similarly, H_S_/D_S_ correlated positively with Pn and Ci (Table 1). These results indicated that there was an intrinsic relationship between these two sets of traits.

**Table 1:** Correlation analysis between F2 seedling morphological and photosynthetic traits

Traits	Tr	Gs	Pn	Ci	WUE
H_S_	0.383	0.412	0.467*	0.662**	-0.646**
D_S_	0.554*	0.527*	0.084	0.208	-0.234
H_S_/D_S_	0.028	0.069	0.459*	0.468*	-0.439

*, significant (*P* < 0.05); **, highly significant (*P* < 0.01); H_S_, seedling height; D_S_, seedling ground diameter; H_S_/D_S_, height/diameter ratio; Tr, transpiration rate; Gs, stomatal conductance; Pn, photosynthetic rate; Ci, intercellular CO_2_ concentration; WUE, water use efficiency

### Variation and correlation of phenotypic traits in the 19 F2 families at the full fruit stage

**Fig. 2:**
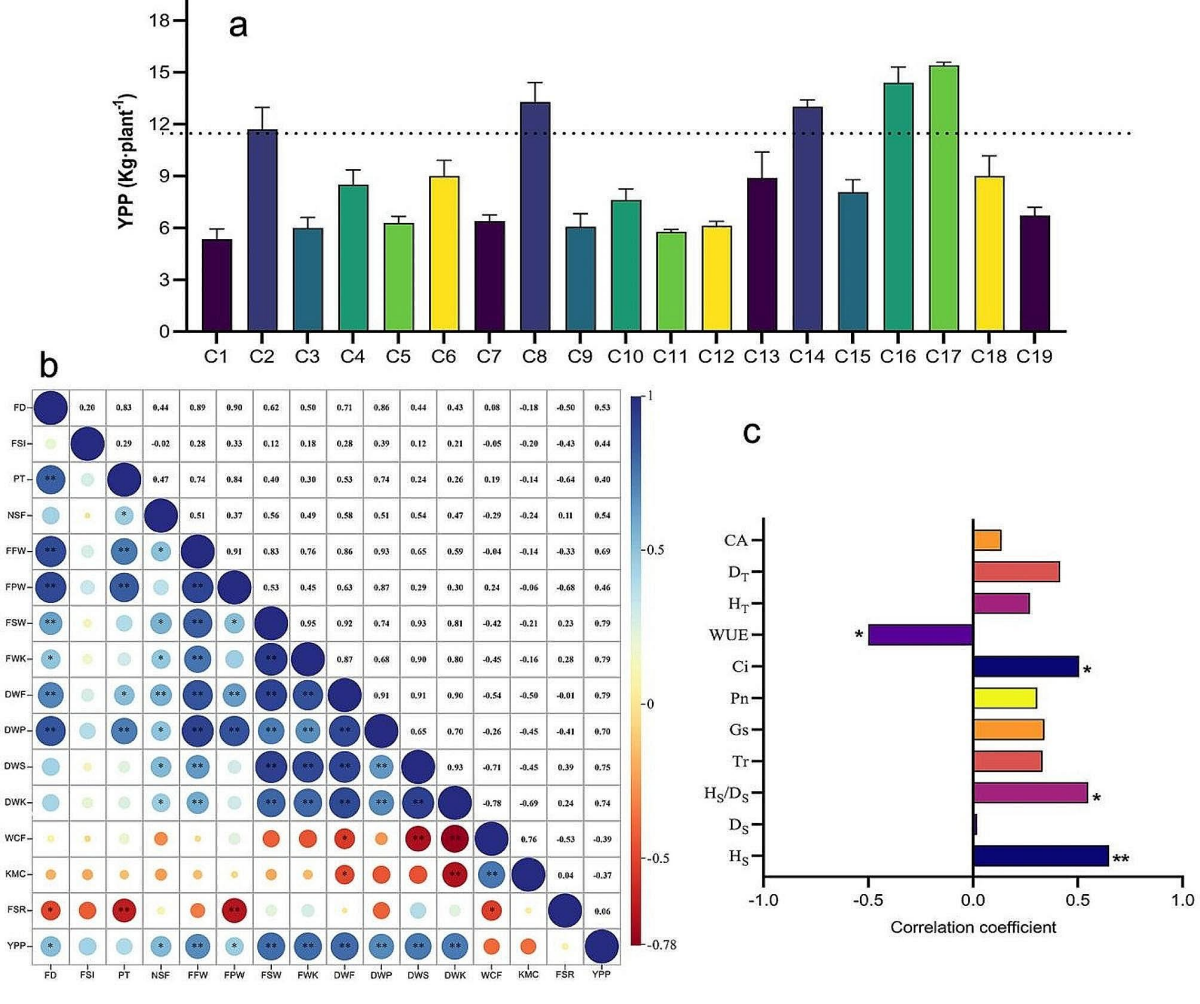
The 19 F2 families yield per plant (YPP) and its correlation with seedling and full fruit stage traits. (**a**) Average YPP of the 19 F2 families. (Dotted lines representing yields equal to more than 30% of the average). (**b**) Correlation between yield traits at full fruit stage. (FD, fruit diameter; FSI, fruit shape index; PT, pericarp thickness; NSF, number of seeds per fruit; FFW, single fresh fruit weight; FPW, fresh pericarp weight; FSW, fresh seed weight; FWK, fresh weight of kernel; DWF, dry weight of fruit; DWP, dry weight of pericarp; DWS, dry weight of seed; DWK, dry weight of kernel; WCF, water content of fruit; KMC, dry weight of seed; FSR, fresh seed rate). (**c**) Correlation between YPP and growth traits at seedling and full fruit stage. (CA, crown area of the tree; D_T_, ground diameter of the tree; H_T_, height of the tree; WUE, water use efficiency; Ci, intercellular CO_2_ concentration; Pn, photosynthetic rate; Gs, stomatal conductance; Tr, transpiration rate; H_S_/D_S_, height/diameter ratio; D_S_, seedling ground diameter; H_S_, seedling height). * and ** *P* < 0.05 and *P* < 0.01 level, respectively

The studied 20 traits produced CV values ranging from 9.65 to 46.23%, among which plant morphological traits, including H_T_, D_T_ and CA, showed CV values ranging between 10.66 and 18.01%, with CA having the largest value (Table S2). Yield traits produced CV values ranging from 9.65 to 46.23%, with FSI showing the smallest value (9.65%), while the remaining traits (NSF, FPW, FSW, DWF, DWP, DWS, DWK, and YPP) had CV values greater than 30% (Table S2). These high CV values, once again, indicate that the 19 F2 families captured abundant variation at full fruit stage, specifically for yield traits. Furthermore, the absolute values of skewness and kurtosis of these traits were less than 3, indicating the presence of continuous normal distribution (Table S2). Selecting those families with 30% higher than the average yield, resulted in identifying 5 F2 families of excellent high-yield with C17 > C16 > C14 > C8 > C2 (Fig. 2a).

Correlation analysis of yield traits at the full fruit stage showed that YPP was significant and positively correlated with FD and NSF, and highly significant and positively correlated with FFW, FSW, FWK, DWF, DWP, DWS, and DWK (Fig. 2b). FFW was positively correlated with DWK, DWS, DWP, DWF, FWK, FSW, FPW, PT, and FD, indicating that yield traits interacted with each other and were coordinated to form the yield per plant. Further analysis of the correlation between YPP and growth traits at the seedling and full fruit stages, showed that YPP was highly significant and positively correlated with H_S_, significant and positively correlated with H_S_/D_S_ and Ci, and negatively correlated with WUE. However, there was no significant correlation between YPP and growth traits at the full fruit stage (Fig. 2c).

### ISSR genetic diversity and genetic cluster analysis

The 12 ISSR primers produced a total of 106 loci, of which 104 were polymorphic. The number of amplified loci per primer ranged between 5 and 14, with average amplification loci of 8.83 and average polymorphic loci of 8.67 (Table 2). PPB ranged from 85.71 to 100%, with an average of 98.21%. The diversity levels varied among the studied primers across the 19 F2 families with average *H* and *I* of 0.3246 and 0.4920, respectively, indicating that these primers reflected the genetic polymorphism among the 19 F2 families.

**Table 2:** Genetic diversity parameters of ISSR markers in the 19 *C. camellia* F2 families

Primer no.	NAL	NPL	PPB	H	I
815	9	9	100	0.2241	0.3661
820	12	12	100	0.2493	0.4046
821	8	8	100	0.3837	0.5698
825	8	8	100	0.2853	0.4398
827	8	8	100	0.3227	0.4892
835	14	13	92.86	0.2802	0.4315
844	7	6	85.71	0.3514	0.5118
845	12	12	100	0.3112	0.4842
856	6	6	100	0.3306	0.5096
858	9	9	100	0.3226	0.4919
865	5	5	100	0.4809	0.6738
873	8	8	100	0.3532	0.5322
Total	106	104	―	―	―
Average	8.83	8.67	98.21	0.3246	0.492

NAL, number of amplified loci; NPL, number of polymorphic loci; PPB, percentage of polymorphic bands; *H*, Nei’s genetic diversity index; *I*, Shannon’s Information index

**Fig. 3:**
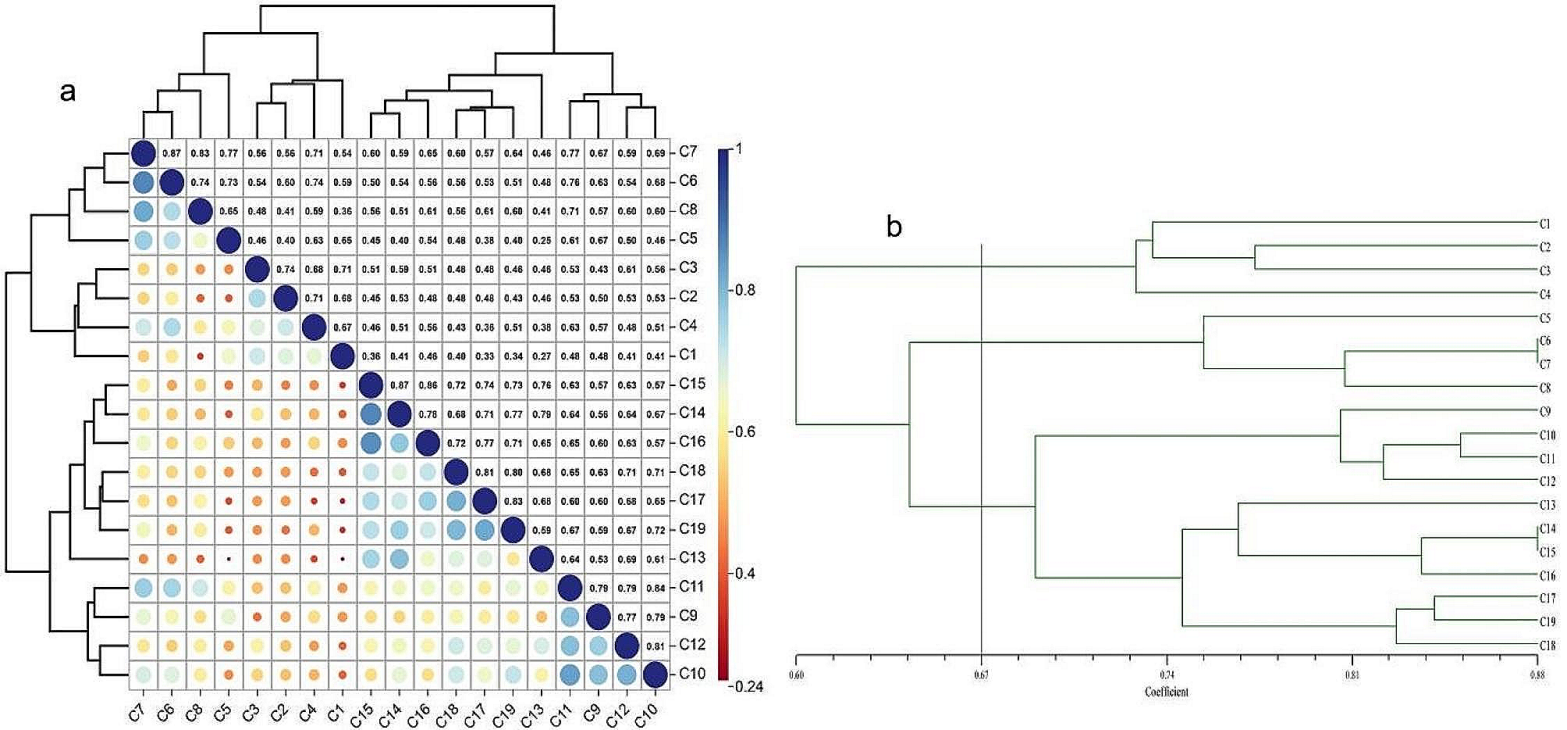
Genetic similarity (**a**) and clustering (**b**) of 19 F2 families

The genetic similarity among the 19 F2 families ranged from 0.25 to 0.87, with an average of 0.67 (Fig, 3a). Using genetic distance of 0.67 as a threshold, the 19 F2 families were divided into three groups (P1, P2 and P3) with different compositions (P1: C1 - C4; P2: C5, - C8; and P3:C9 - C19) (Fig. 3b).

### Genetic structure analysis of 19 F2 families

**Fig. 4:**
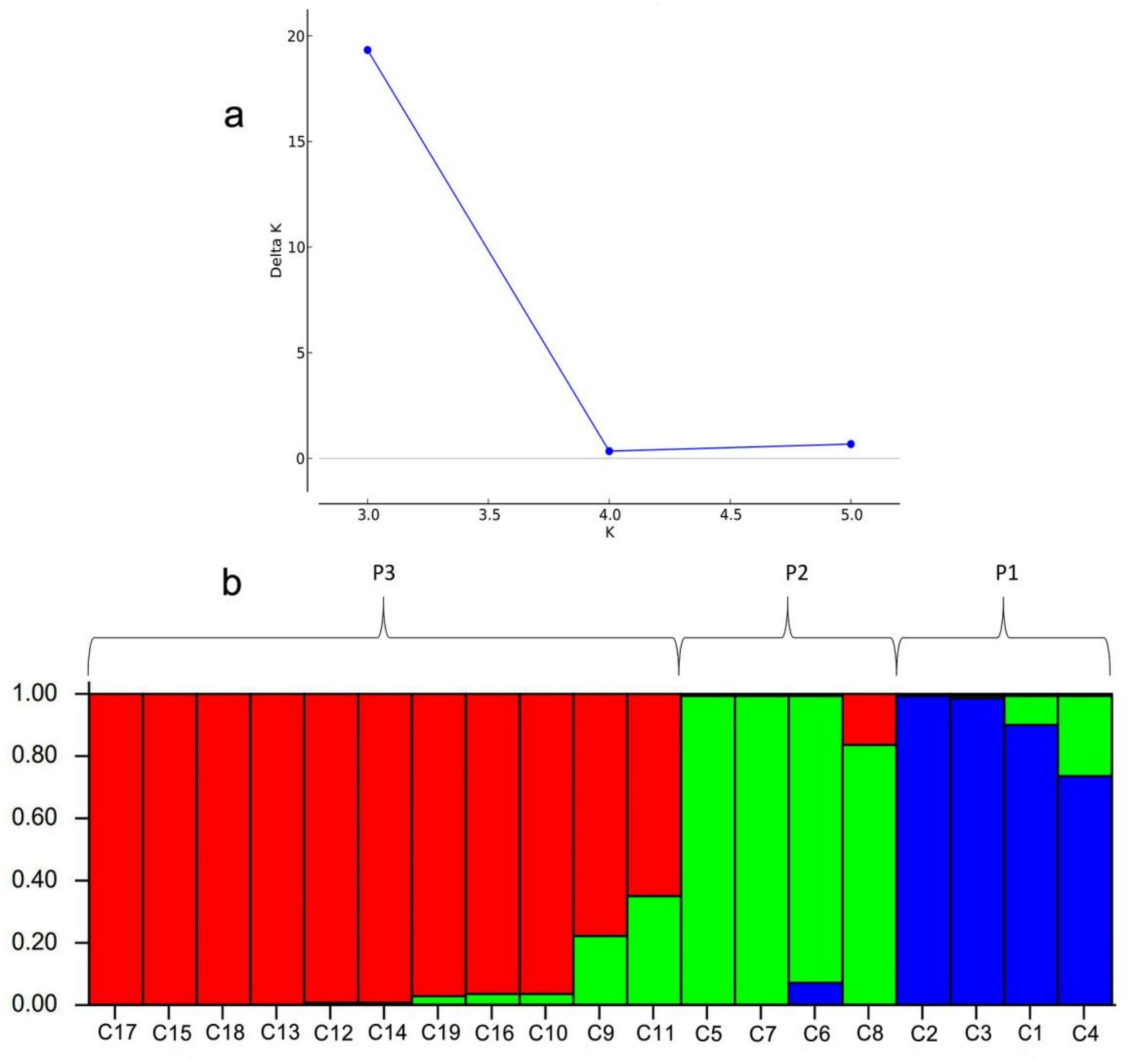
Genetic structure of 19 F2 *C. oleifera* families: (**a**) Variation trend of K and ∆K, (**b**) Population genetic structure at K = 3

Population genetic structure analysis indicated that when K = 3, ∆K showed the maximum peak value (Fig. 4a), thus, the 19 F2 families can be divided into 3 subgroups similar to that obtained from the clustering analysis above (Fig. 4b).

### Association between ISSR markers and characters at seedling traits

Association between ISSR markers and the 8 seedling phenotypic traits yield five (D_S_, H_S_, WUE, H_S_/D_S_, and Tr) produced highly significant associations with 7 loci (Table 3). These seven out of the 106 loci showed association with seedling phenotypic traits (6.60%) (Table 2). The percent variance (*R*^2^) of these associations ranged from 27.63 to 59.54% (Table 3). Among the seven significant associated loci, 820-7 was associated with both H_S_ and H_S_/D_S_, and their *R*^2^ exceeded 30% (Table 3).

**Table 3:** Association/correlation between ISSR markers and seedling phenotypic traits

Traits	Associated loci	*P*-value	*R* ^2^
D_S_	873-4	0.0078	27.63
H_S_	820-7	0.0005	39.87
WUE	815-3	0.0081	22.69
	815-6	0.0081	22.69
H_S_/D_S_	820-7	0.0002	59.54
	820-8	0.0014	48.44
Tr	845-7	0.0090	25.82

D_S_, seedling ground diameter; H_S_, seedling height; WUE, water use efficiency; H_S_/D_S_, height/diameter ratio; Tr, transpiration rate; *R*^2^, % variance

### Association between ISSR markers and characters at the full fruiting stage

Association between ISSR markers and the 20 phenotypic traits at the full fruit stage indicted that a total of 13 loci were significantly associated with 16 phenotypic traits (FH, FD, FSI, PT, NSF, FFW, FPW, FSW, FWK, DWF, DWP, DWS, DWK, WCF, FSR and YPP) (Table 4). Thirteen out of the 106 loci showed association with phenotypic traits at the full fruit stage (12.26%) (Table 2). The percent variance *R*^2^ at the full fruit stage ranged from 12.37 to 58.42%. With the exception of PT (*R*^2^ = 12.37%), the *R*^2^ values of the remaining traits were greater than 20% (Table 4).

**Table 4:** Association/correlation between ISSR markers and phenotypic traits at the full fruit stage

Traits	Associated loci	*P*-value	*R* ^2^
FH	845-9	0.0005	34.82
	858-2	0.0005	35.02
FD	844-9	0.0022	20.76
	858-2	0.0005	24.73
FSI	845-9	0.0001	58.42
PT	844-9	0.0060	12.37
NSF	815 − 12	0.0076	27.40
	827-3	0.0064	28.27
FFW	827-5	0.0009	25.76
	858-2	0.0017	23.97
FPW	858-2	0.0008	24.36
FSW	815-5	0.0066	29.55
	835 − 12	0.0002	45.59
	858-3	0.0091	27.72
FWK	835 − 12	0.0010	45.57
DWF	835 − 12	0.0003	41.87
	845 − 11	0.0047	29.81
DWP	845-9	0.0073	22.61
DWS	835 − 12	0.0001	54.42
	845 − 11	0.0051	35.97
	858 − 10	0.0055	35.49
	858-3	0.0036	38.05
DWK	815 − 11	0.0029	39.64
	835 − 12	0.0002	52.70
	858 − 10	0.0060	35.23
WCF	815 − 11	0.0043	37.34
	835 − 12	0.0066	34.62
FSR	858-3	0.0097	27.41
YPP	835-3	0.0082	29.82
	835 − 12	0.0078	30.08

*R*^2^, % variance; FH, fruit height; FD, fruit diameter; FSI, fruit shape index; PT, pericarp thickness; NSF, number of seeds per fruit; FFW, single fruit fresh weight; FPW, pericarp fresh weight; FSW, seed fresh weight; FWK, fresh weight of kernel ; DWF, dry weight of fruit; DWP, pericarp dry weight; DWS, dry weight of seed; DWK, dry weight of kernel; WCF, water content of fruit; KMC, kernel moisture content; FSR, fresh seed rate; YPP, yield per plant

### Associated loci in the F2 populations

According to the association analyses results (above), 19 ISSR loci showed significant association with both the seedling and full fruit stages traits, and these significant loci were identified in the F2 generation (Table S3), additionally the polymorphism ratio of significantly associated loci per F2 family (PSL) ranged from 10.53 to 47.37% (Table 5). Nine significantly associated loci (835 − 12, 858-2, 858-3, 827-5, 820-7, 815-5, 815-6, 845-9 and 845 − 11) had higher polymorphism ratio of partial female (PRF) than partial male (PRM) (Table S3), indicating that they followed partial maternal inheritance. While polymorphism ratio of partial male (PRM) of two loci (827-3 and 815-6) were higher than PRF, indicating that they followed partial paternal inheritance (Table S3). The PRF of C2, C10, C13, C14 and C15 were higher than PRM, indicating that these 5 F2 families followed partial maternal inheritance. The PRM of C5, C11, C16 and C18 was higher than PRF, indicating that the four F2 families followed partial paternal inheritance (Table 5).

**Table 5:** Deviation analysis of significantly associated loci in the 19 hybrid F2 generation

Diallel family ID	SLM	SLP	NSL	PSL	PRF	PRM
C1	1	1	3	15.79	0.33	0.33
C2	3	2	5	26.32	0.60	0.40
C3	1	1	3	15.79	0.33	0.33
C4	1	1	2	10.53	0.50	0.50
C5	1	2	2	10.53	0.50	1.00
C6	1	1	2	15.79	0.50	0.50
C7	1	1	2	15.79	0.50	0.50
C8	1	1	5	26.32	0.20	0.20
C9	2	2	5	26.32	0.40	0.40
C10	2	1	4	21.05	0.50	0.25
C11	1	2	3	15.79	0.33	0.67
C12	1	1	3	15.79	0.33	0.33
C13	5	0	9	47.37	0.56	0.00
C14	3	0	5	26.32	0.60	0.00
C15	3	1	5	26.32	0.60	0.20
C16	0	1	3	15.79	0.00	0.33
C17	0	0	2	10.53	0.00	0.00
C18	0	1	4	21.05	0.00	0.25
C19	0	0	3	15.79	0.00	0.00

SLM, number of significant association locus within the F2 families that were the same as the mother; SLF, number of significant association locus in the hybrid combination that are the same as the father; NSL, number of significantly associated loci in the F2 families; PSL, polymorphism ratio of significantly associated loci per F2 family; PRF, polymorphism ratio of partial female; PRM, polymorphism ratio of partial male

There were significantly associated loci were detected in five F2 high-yield families (C2, C8, C14, C16 and C17) (Table 6). Each of these families showed significant association with specific loci (Family C2 with 835-3, 844-9, 858-2, 873-4, and 815; family C8 with 835-3, 858-3, 858 − 10, 845-7 and 845 − 11; family C14 with 858-3, 827-3, 820-7, 815 − 11 and 845 − 11; family C16 had 835 − 12, 827-3 and 815 − 11 and family C17 with 815 − 11 and 815 − 12 (Table 6).

**Table 6:** Significant ISSR loci associated in the 5 F2 high-yield *C. oleifolia* families

SAL	C2	C8	C14	C16	C17
835-3	√	√	-	-	-
835 − 12	-	-	-	√	-
844-9	√	-	-	-	-
858-2	√	-	-	-	-
858-3	-	√	√	-	-
858 − 10	-	√	-	-	-
827-3	-	-	√	√	-
827-5	-	-	-	-	-
820-7	-	-	√	-	-
820-8	-	-	-	-	-
873-4	√	-	-	-	-
815-3	-	-	-	-	-
815-5	-	-	-	-	-
815-6	√	-	-	-	-
815 − 11	-	-	√	√	√
815 − 12	-	-	-	-	√
845-7	-	√	-	-	-
845-9	-	-	-	-	-
845 − 11	-	√	√	-	-

SAL, significantly associated loci

Furthermore, these five parents showed polymorphism ratio of significantly associated loci per family (PSL) ranging from 5.26 to 31.58%. These parents showed significant association with various loci (Parent 27 − 5 with 835-3, 858-2, 815-6 and 845-9; parent 41 − 4 with835-12 and 845 − 11; parent 46 − 2 with 835-3, 835 − 12, 858 − 10 and 815-6; parent 51 − 2 with 858-3, 827-3, 827-5, 820-7, 815-5 and 845 − 11 and parent 56 − 6 with 858-3) (Table 7).

**Table 7:** Significant ISSR loci associated in the 5 *C. oleifolia* parents

SAL	Parents				
	27 − 5	41 − 4	46 − 2	51 − 2	56 − 6
835-3	√	-	√	-	-
835 − 12	-	√	√	-	-
844-9	-	-	-	-	-
858-2	√	-	-	-	-
858-3	-	-	-	√	√
858 − 10	-	-	√	-	-
827-3	-	-	-	√	-
827-5	-	-	-	√	-
820-7	-	-	-	√	-
820-8	-	-	-	-	-
873-4	-	-	-	-	-
815-3	-	-	-	-	-
815-5	-	-	-	√	-
815-6	√	-	√	-	-
815 − 11	-	-	-	-	-
815 − 12	-	-	-	-	-
845-7	-	-	-	-	-
845-9	√	-	-	-	-
845 − 11	-	√	-	√	-
PSL %	21.05	10.53	21.05	31.58	5.26

SAL, significantly associated loci; PSL, polymorphism ratio of significant association locus per F2 family

## Discussion

### Rich genetic diversity laid the foundation for association analysis

For natural variation, association analysis can detect genetic variation extensively with high resolution. Plant phenotypic variation is the embodiment of genetic material diversity [31, 32]. There was a large amount of variation between plant growth and yield traits of *C. oleifolia* F2 generation at both the seedling and full fruit stages, with coefficient of variation ranging between 9.65 and 46.73%, indicating high degree of dispersion, and confirming that the F2 generation had excellent genetic basis for improvement [33, 34]. Among the yield traits, the CV of NSF was the largest (42.23%), while FSI was the smallest (9.65%), observations similar to those previously reported studies [35]. This may be related to the breeding goal of high yield, which promotes strong separation of yield traits in F2 generation by crossing F1 siblings [36]. The CV of plant morphology indices were the smallest, followed by photosynthetic physiological traits at the seedling stage, and the largest were for yield traits at the full fruit stage, indicating that *C. oleifolia* F2 generation population was suitable for the implemented association analysis of yield trait and the selection of high-yield family [34, 36].

Genetic diversity analysis also relies on molecular markers, as DNA markers are not affected by environmental conditions and plant development stage (i.e., neutral) [15, 37]. The PPB of the ISSR primers in the studied F2 hybrid combinations ranged between 85.71 and 100% with an average of 98.21%, higher values than those reported between germplasm resources (92.56%) and clones (68.60-87.96%) [38, 39]. The average *H* and *I* were 0.3246 and 0.492, respectively, indicating that the *C. oleifolia* F2 generation had rich genetic diversity and broad genetic basis [40]. Additionally, the ISSR molecular marker had good polymorphic expression, that can fully distinguish among the F2 hybrid combinations on the molecular level for association analysis [40, 41]. Therefore, the rich genetic diversity present in the *C. oleifolia* F2 hybrid combinations, provided powerful information for the genetic population structure analysis, and can greatly benefit the association analysis efficiency.

### High yield molecular markers detected by association analysis

Association analysis is based on the presence of linkage disequilibrium between marker loci and causal genes underpinning target traits. It is necessary to understand population’s linkage disequilibrium before embarking on association analysis [42, 43]. Our genetic structure was completely consistent with the results of cluster analysis in *C. oleifolia* F2 generation population, indicating that the polymorphic loci detected by ISSR were in linkage disequilibrium with functional loci [44], and would not lead to the generation of pseudo-association [45]. The genetic variation of *C. oleifolia* F2 hybrid combinations have enhanced the degree of polymorphism and in turn increased the resolution in linkage disequilibrium detection [46, 47]. The number of the test population and marker is also one of the factors affecting linkage disequilibrium [48]. Significant associated locus with test value of 0.0001 could be detected, indicating that the F2 population composed of 597 individuals and 12 ISSR markers has enough sensitivity to detect the observed associations in the studied F2 population [49].

It is generally believed that continuous or intermittent variables of biological phenomena in the natural state, conform to the normal distribution [50]. The skewness and kurtosis of all the studied phenotypic traits in the F2 population were less than 3, indicating that they followed continuous normal distributions and quantitative genetic characteristics, and can be used for association analysis [51, 52]. However, the kurtosis of FH, FFW and YPP were higher than 2, which may be due to the fact that they are important economic traits related to the high-yield breeding process of *C. oleifolia* [53], and were subjected to selection pressure resulting in the observed skewed distribution [54]. The 19 ISSR loci were significantly associated with the 21 phenotypic traits, and the number of associated loci accounted for 17.92% of the total studied loci. This is slightly higher than that obtained from RNA-Seq technology in a GWAS study that accounted for 16.56% of the total SNPs used [27, 28], indicating that ISSR, as a dominant gene marker, can efficiently detect associated loci [55]. Therefore, it was feasible and efficient to conduct association analysis of *C. oleifolia* F2 population based on ISSR molecular markers.

Obtaining excellent molecular markers tightly linked to target traits is a prerequisite for developing marker-assisted breeding [56]. Previous studies believed that the accuracy of genome-wide selection can only be ensured if the percent contribution (*R*^2^) is greater than 20% [57, 58]. The *R*^2^ of *C. oleifolia* phenotypic traits were greater than 20%, except for PT, indicating the association analyses of *C. oleifolia* F2 generation were accurate. Among the loci with multiple effects, 820-7 at seedling stage and 835 − 12 at full fruit stage, had *R*^2^ greater than 50% and the largest number of significant associated traits. The 858-3 locus was highly significant association with seed fresh weight (FSW), dry weight of seed (DWS) and fresh seed rate (FSR), all with *R*^2^ greater than 20%, which determine the seed yield of *C. oleifolia*. These three loci were present in excellent high-yield families (858-3 in C14 and C8; 835 − 12 in C16; 820-7 in C14). Additionally, 815 − 11 locus was present in the top three excellent high-yield families (C14, C16 and C17). Thus, 815 − 11, 820-7, 858-3 and 835 − 12 loci represent excellent high-yield associated ISSR markers.

### Molecular marker assisted *C. Oleifolia* breeding

It is generally believed that the selected molecular markers associated with the target traits can be used for their detection at any stage of plant growth and development [56, 59]. The correlation between traits at different developmental stages is helpful to the selection and utilization in practical breeding, improve breeding efficiency, and providing a reference for early selection in forest trees [4, 60]. *C. oleifolia* yield traits interact and coordinate with each other, to ultimately affecting the yield per plant (YPP) [10, 61]. Here, we observed 10 yield traits that were positively and significantly correlated, among which the most significant correlation was fresh weight of kernel (FWK). Oil yield of *C. oleifolia* is determined by YPP and oil content of fresh fruit [62], and previous studies suggested that FWK are positively and significantly correlated with oil content of fresh fruit [61]. Therefore, FWK was an important trait for early identification of high-yield *C. oleifolia*. In breeding practice, the heavier the fresh kernel of *C. oleifolia*, the higher the yield and the greater the rate of improvement. The tightness between growth traits and ISSR markers at the seedling stage was higher than that of yield traits at full fruit stage. It may be that yield phenotypic traits of adult tree were more susceptible to environmental influences than growth phenotypic traits of seedling [63]. So, the yield associated loci obtained at seedling stage were more accurate than those at full fruit stage. The YPP was positively and significantly correlated with height (H_S_), indicating that the taller the seedling, the more likely the cultivated *C. oleifolia* plants will produce high yield, similar observations were reported in other studies [25]. In summary, early selection of high-yield superior plant of *C. oleifolia* can be achieved, by measuring fresh weight of kernel and seedling height, which can be captured with the presence of these associated marker loci (815 − 11, 820-7, 835 − 12 and 858-3).

*C. oleifolia* traditional breeding often takes ten years or even decades to breed a good variety [24, 61]. The primary problem of *C. oleifolia* low breeding efficiency, can be enhanced through selecting suitable parents and capitalize on their specific combining ability [53, 61]. In the studied F2 generation, there were excellent high-yield combinations (e.g., C2, C8, C14, C16 and C17), and all produced more than twice the national high yield breeding standard [64]. Among these selections, C2 and C8 had the most distant relation, and C2 expressed partial maternal inheritance, thus it is selected as mother and C8 as father which is expected to produce higher yield *C. oleifolia* variety [65]. Additionally, while C16 and C17 had close distant relation with the lowest polymorphism rate, if they are selected with C16 as a male parent (as it possesses partial paternal inheritance and the 835 − 12 locus) and C17 as female parent (as it possesses the 815 − 11 locus), this combination can accelerate the introduction of high-yield through the association of high-yield causal genes with 815 − 11 and 835 − 12 loci, and ultimately reducing the breeding workload [66]. Furthermore, the 815 − 11 locus is only present in the top three excellent high-yield F2 families (C14 and C16 with 51 − 2 as a male parent, and C17 with 51 − 2 as a female parent), not in any of the parents of excellent high-yield families, which may be a recessive gene, and produced by hybridization and recombinant with 51 − 2 as parent [67]. Additionally, 51 − 2 had the highest polymorphism ratio of significantly associated loci (PSL) in the F1 generation and the excellent high-yield associated locus of 858-3 and 820-7. The C14 had the highest PSL, and partial maternal inheritance. The backcross (C14 as a female parent and 51 − 2 as male parent) can improve the efficiency of introducing high-yield associated loci of 815 − 11, 858-3 and 820-7, especially 815 − 11. So, to increase the frequency of high-yield gene, specific parental combinations were identified based on their partial maternal or maternal inheritance which can improve the breeding selection accuracy of *C. oleifolia* [68, 69].

## Conclusions

High coefficient of variation, extent of genetic variation, of 28 traits seedling and full fruit stages of *C. oleifolia* 19 F2 generation hybrids was observed. Five hybrid combinations (C2, C8, C14, C16 and C17) showed great propensity for excellent high-yield. A total of 106 alleles were obtained from 12 ISSR primers, and average polymorphic ratio of 98.21%. Population structure and cluster analysis showed that F2 generation population could be divided into 3 subgroups. Through association analysis, a total of 19 loci were associated with 21 traits at seedling and full fruit stages with *R*^*2*^ ranging from 12.37 to 59.54%. The number of associated loci accounted for 17.92%, and four loci (815 − 11, 835 − 12, 820-7 and 858-3) were selected as excellent high-yield ISSR markers. Fresh weight of kernel and seedling height can be used as high-yield selection indexes. So, early selection of high-yield hybrid superior variety can be realized by screening for greater fresh kernel weight and taller 1-year old seedlings, and identifying these 4 excellent high-yield ISSR markers. Specific parental combinations were identified based on their partial maternal or maternal inheritance and ISSR markers associated with high-yield. The use of molecular assisted breeding can improve the high-yield selection accuracy and efficiency of *C. oleifolia*.

## Materials and methods

### Materials

The research material for this study originated from: (1) in 1973, 7 *C. oleifolia* phenotypically superior individuals (parents: Min 43, Min 49, Min 50, Min 53, Min 54, Min 56, and Min 62) were selected throughout the Fujian Province, and were used to establish a clonal experimental forest at the Tongkou forest farm in Fuzhou City, Fujian province, China; (2) in 1985, these 7 parents produced 5 F1 generation full-sib families through hybridization and were planted at the Tongkou forest farm; (3) in 2007, after testing these F1 families over 5 consecutive years, 5 phenotypically superior individuals (27 − 5, 41 − 4, 46 − 2, 51 − 2, and 56 − 2), were selected to produce complete 5 × 5 diallel mating design that resulted in 19 F2 families (C1, C2, C3, …, C19). Among them, 56 − 2 as the mother and 41 − 4 as the father had incompatibility; (4) in 2008, seed from these 19 F2 diallel crosses (C1, C2, C3, …, C19) were used to produce seedlings for further testing; (5) in 2009, these seedlings were planted in a randomized complete block design with three 19 × 10 row plots in Tongkou forest farm with 30 plants per diallel crosse, and a total of 570 plants; and (6) in 2020, the F2 families’ offspring were in their full fruit stage, then fruits were harvested, and tree yield and growth and fruit traits were measured over 3 consecutive years. See Table 8; Fig. 1 for the genetic material identification and their parental origins.

**Table 8:** The original 7 parents 5 F2 parents, and their 19 diallel crosses in F2 generation

Number	♀x♂	Number	♀x♂	Number	♀x♂
27 − 5	Min 62×Min 56	C4	27 − 5 × 41 − 4	C12	46 − 2 × 56 − 2
41 − 4	Min 54×Min 43	C5	41 − 4 × 51 − 2	C13	51 − 2 × 46 − 2
46 − 2	Min 50×Min 62	C6	41 − 4 × 46 − 2	C14	51 − 2 × 27 − 5
51 − 2	Min 43×Min 53	C7	41 − 4 × 27 − 5	C15	51 − 2 × 56 − 2
56 − 2	Min 43×Min 49	C8	41 − 4 × 56 − 2	C16	51 − 2 × 41 − 4
C1	27 − 5 × 51 − 2	C9	46 − 2 × 51 − 2	C17	56 − 2 × 51 − 2
C2	27 − 5 × 46 − 2	C10	46 − 2 × 27 − 5	C18	56 − 2 × 46 − 2
C3	27 − 5 × 56 − 2	C11	46 − 2 × 41 − 4	C19	56 − 2 × 27 − 5

### Crossing method

In late November 2006, flowers at the full-bloom stage were selected at the top of branches. Pollen was collected form male parent plants. For female parent plants, flowers were emasculated with tweezers, then isolated with paper bags (15 × 15 cm). In 2–3 days after emasculation, a special mucus was secreted from the stigma, which can be pollenated. 8 days after pollination, followed by petal colour fading and drying of stigma, demarking the time for pollination bags removal. In early November 2007, the pollinated fruit are ripped and harvested. Each combination (C1, C2, C3, …, C19) of crosses yielded 30 fruits.

### Seedlings’ phenotyping

After collecting the fruits of the original 7 parents, 5 F2 parents, and their 19 diallel crosses F2 generation, they were naturally cracked, seeds were collected, and stored in sand over winter. In March 2008, seeds were cultivated for seedlings production.

In March 2010, 30 seedlings were randomly selected for each diallel crosse (570 seedlings from the 19 F2 diallel crosses) were measured for height (H_S_), ground diameter (D_S_), and height/diameter ratio (H_S_/D_S_). From each F2 cross, 5 seedlings were selected, and the third leaf of the shoot was selected from the four cardinal directions to determine their photosynthetic index. Weather permitting, transpiration rate (Tr), stomatal conductance (Gs), photosynthetic rate (Pn), intercellular CO_2_ concentration (Ci), and water use efficiency (WUE) were measured using the GFS-3000 portable photosynthesiser (WALZ, Germany).

### Full fruit stage phenotyping

In early November 2020, trees at the full fruit stage were selected, and the yield per plant (YPP), height of the tree (H_T_), ground diameter of the tree (D_T_), and crown area of the tree (CA) were measured after fruit harvesting. Fifteen fruits were randomly selected from each tree, and fruit height (FH) and diameter (FD) were measured with vernier calipers (accurate to 0.01 mm), and fruit shape index (FSI) was calculated. Then, after measuring single fresh fruit weight (FFW) of 15 fruits with electronic balance (accurate to 0.01 g), pericarp was immediately removed and number of seeds per fruit (NSF) was counted, fresh seed weight (FSW) and fresh pericarp weight (FPW) were determined, and fresh seed rate (FSR) was calculated. Then the seed coat was removed and fresh weight of kernel (FWK) was measured. Pericarp thickness (PT) was measured with vernier caliper. Then the pericarp, seed coat, and seed kernel were placed in a drying oven at 105˚C for green treatment, and then baked to constant weight at 60˚C. The dry weight of pericarp (DWP), seed coat and kernel (DWK) were determined, and dry weight of fruit (DWF), dry weight of seed (DWS), water content of fruit (WCF), and kernel moisture content (KMC) were calculated as follows:

1 $$FSI=\frac{FH}{FD}$$

2 $$FSR=\frac{FSW}{FFW}$$

3 $${DWS}={Dry}\,\,{weight}\, {of}\, {seed}\, {coat} +DWK$$

4 $$DWF=DWS+DWP$$

5 $$WCF=\frac{DWF}{FFW}$$

6 $$KMC=\frac{DWK}{FWK}$$

### Genomic DNA extraction and ISSR primer screening

In March 2010, 1–2 young leaves free of pests and diseases were collected from each seedling. After mixing the F2 diallel crosses, F2 parents and original parents, the leaves were stored at -70℃ until further use. DNA was extracted by the CTAB method and stored at -20˚C. DNA concentration and quality of the 19 diallel crosses and the 5 parents were detected. Absorbed 1ul of completely dissolved DNA stock solution and 1ul of 6×Loading buffer, mixed the two solutions evenly. Then they were sampled in1.5% TAE agar gel with 15,000 bp DNA maker as the control. The gel electrophoresis was conducted in 1×TAE buffer (voltage 150 V, current 120 mA) for 30 min, and placed in ethidium bromide (EB) for 10 min. DNA with clear bands and no obvious degradation, and diluting to 50 times (30 ng/µL), was best for ISSR-PCR amplification. A total of 12 ISSR primers with high polymorphism, good stability and clarity were selected (Table S4).

### ISSR*-*PCR reaction system and DNA amplification

The ISSR reaction system was established as 20 µl (Table S5) using the Master-Cycler 05 gradient PCR instrument (Eppendorf, Germany). PCR hot cap temperature to 105˚C, pre-denaturation was 5 min at 94˚C, denaturation was 45s at 94˚C, annealing temperature was 54˚C, and elongation was 1.5 min at 72 C with 39 cycles, extended at 72˚C for 7 min, and stored at 4˚C. Added 1/6 volume of 6×Loading Buffer into the ISSR-PCR amplification product. Loaded the sample on 1.5% agarose gel, use Maker as the control, with 1×TAE electrophoresis buffer solution at 150 V constant voltage and 120 mA current for 1 h. When the Loading Buffer indicator reached the bottom of the gel, electrophoresis was stopped, and the gel was stained in EB (final concentration is 0.5ul/ml) for 10–15 min and imaged under ultraviolet light of the gel imaging system. The 31 genetic groups (the original 7 parents, 5 F2 parents, and their 19 diallel crosses F2 generation) were amplified by screened ISSR primers (Fig.S1).

### Genetic parameter estimation and cluster analysis

Max and min values, range, mean, standard deviation (SD), skewness, kurtosis, coefficient of variation (CV), and correlations of *C. oleifolia* 28 phenotypic characters at the seedling and full fruit stages were analyzed using SPSS26.0 software [70]. When CV < 10%, characters showed low variation, 10 < CV < 20% moderate variation, 20 < CV < 30% high variation, and CV > 30% strong variation [33]. The amplified products were interpreted by visual inspection, and an ISSR molecular label 0/1 matrix was established for each band with or without a value of 1 and 0, respectively. Number of amplification loci (NAL), number of polymorphic loci (NPL), percent of polymorphic band (PPB), and genetics similarity (*Gs*) were calculated.

7 $$PPB=\frac{NPL}{NAL}\times 100\%$$

8 $${Gs}\,=\,{N}_{ij}/({N}_{i}\,+\,{N}_{j})$$

where, *N*_*ij*_ representing the same number of bands between two F2 families, and *N*_*i*_ and *N*_*j*_ representing the number of bands in each of the two F2 families [71].

POPGENE 1.32 software was used to calculate Nei’s genetic diversity index (*H*) and Shannon’s Information index (*I*) [72]. UPGMA method of NTsys2.10e software was used to calculate genetic distance for cluster analysis, and SAHN in clustering program was used to obtain cluster graph [73].

9 $$H=1-\sum {P}_{i}^{2}$$

where, *P*_*i*_ is the frequency of an amplified product, which was used to reflect the abundance and uniformity of alleles among F2 families.

10 $$I=\sum {P}_{i}\,\text{ln}\,{P}_{i}$$

where, *P*_*i*_ is the frequency of occurrence of a certain genotype in a population, which mainly reflects the richness of phenotypic diversity [40].

### Population genetic structure analysis

The software Structure 2.3.4 was used to analyze the population genetic structure of the tested materials, and the predicted population number K was set to 2 ∼ 6 [74, 75]. Length of burn-in period of Markov Chain Monte Carlo (MCMC) was set to 100,000. Each K value was repeated 10 times to calculate the corresponding Q value for each combination. The optimal number of subgroups is determined by calculating the optimal K value. ΔK was calculated according to ln*P*(*D*), and the K value at the maximum. ΔK value was taken as the best subgroup number, and the probability (Q value) of genotype belonging to subgroup K of all the tested materials was calculated synchronically. The online program Structure harvester (http://taylor0.biology.ucla.edu/struct/) was used [75].

### Association analysis of phenotypic traits and markers

Association analysis and genetic similarity results were analyzed using SPSS26.0 software [70]. The general linear model (GLM) of TASSEL-5.0 software was used to correlate phenotypic data in different periods with the Q value of each F2 family as the covariate, and the loci associated with marker variation and phenotypic traits were detected under *P* < 0.01. The rate of explanation on the phenotype variance of related markers (i.e., % variance) (*R*^2^) were calculated following Ping et al. [76].

### **Deviation analysis of significantly association markers**

In the 19 F2 families and their 5 parents, ISSR bands with very significant association with phenotypic traits were marked as “√”, and those without any significant association or missing or fuzzy stripe types were marked as “-”. In the 19 F2 families, if the family has the same bands as the mother were labeled “♀” and the same bands as the father were labeled “♂”. The polymorphism ratio of significantly associated loci per F2 family (PSL), polymorphism ratio of partial female (PRF), and polymorphism ratio of partial male (PRM) were calculated as follows:

11 $$PSL\%=\frac{NA}{NAT}\times 100$$

where, NA is the number of significantly associated locus within the F2 family and NAT showing the number of significantly associated locus in the F2 population.

12 $$PRF=\frac{SLM}{NSL}$$

13 $$PRM=\frac{SLF}{NSL}$$

where, SLM is the number of significantly associated loci within the F2 family that were the same as the mother, NSL is the number of significantly associated loci within the F2 family, SLF is the number of significantly associated loci in the F2 family that are the same as the father [77, 78].

### Electronic supplementary material

Below is the link to the electronic supplementary material.

Supplementary Material 1

## Data Availability

The original contributions presented in the study are included in this article. Further inquiries can be directed to the corresponding author.

## Abbreviations

H_S_: Seedling height
D_S_: Seedling ground diameter
H_S_/D_S_: Height/diameter ratio
Tr: Transpiration rate
Gs: Stomatal conductance
Pn: Photosynthetic rate
Ci: Intercellular CO_2_ concentration
WUE: Water use efficiency
SD: Standard deviation
CV: Coefficient of variation
YPP: Yield per plant
FD: Fruit diameter
FH: Fruit height
FSI: Fruit shape index
PT: Pericarp thickness
NSF: Number of seeds per fruit
FFW: Single fresh fruit weight
FPW: Fresh pericarp weight
FSW: Fresh seed weight
FWK: Fresh weight of kernel
DWF: Dry weight of fruit
DWP: Dry weight of pericarp
DWS: Dry weight of seed
DWK: Dry weight of kernel
WCF: Water content of fruit
KMC: Kernel moisture content
FSR: Fresh seed rate
CA: Crown area of the tree
D_T_: Ground diameter of the tree
H_T_: Height of the tree
NAL: Number of amplified loci
NPL: Number of polymorphic loci
PPB: Percentage of polymorphic bands
H: Nei’s genetic diversity index
I: Shannon’s Information index
SLM: Number of significant association locus in the hybrid combination that is the same as the mother
SLF: Number of significant association locus in the hybrid combination that is the same as the father
NSL: Number of significantly associated loci in the F2 families
PSL: Polymorphism ratio of significantly associated locus per F2 family
PRF: Polymorphism ratio of partial female
PRM: Polymorphism ratio of partial male
SAL: Significantly associated loci
PSL: Polymorphism ratio of significant association locus per F2 family
