# Supplementary material for: High-yield hybrid breeding of Camellia oleifolia based on ISSR molecular markers
Source: BMC Plant Biol. 2024 Jun 8;24:517. doi: 10.1186/s12870-024-05218-x (PMC11162053; doi:10.1186/s12870-024-05218-x)
Supplement: Supplementary file 1 — Supplementary Material 1 [file 12870_2024_5218_MOESM1_ESM.pdf]

Supplementary materials:

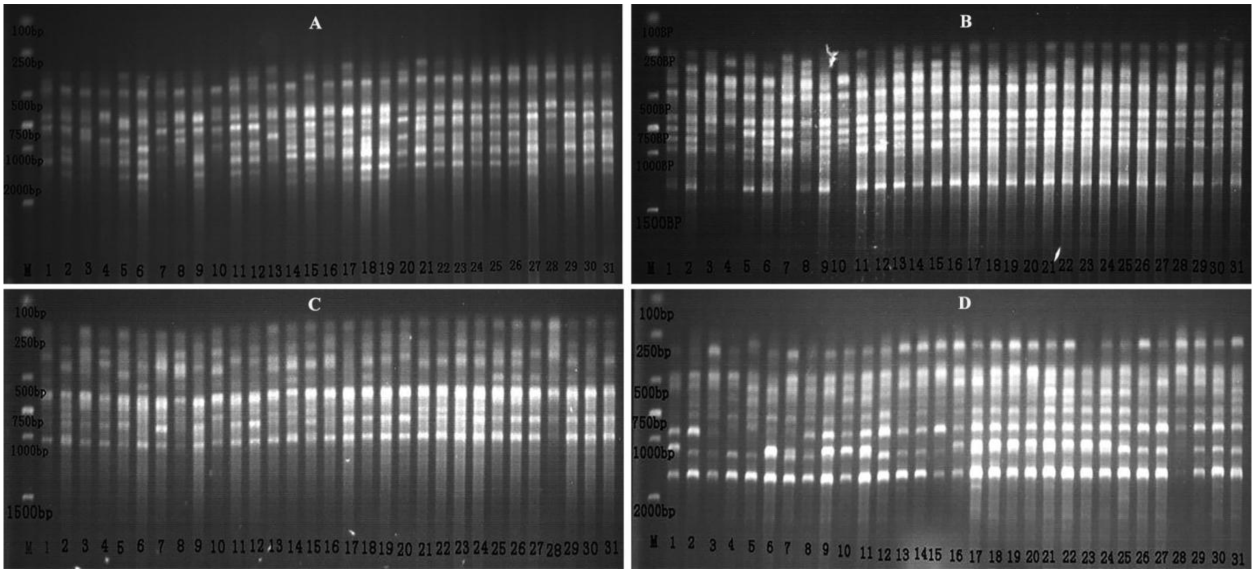

Fig.S1 ISSR amplification of the original 7 parents, 5 F2 parents, and their 19 diallel crosses in F2 generation by Some primers. A: ISSR820 primer; B: ISSR856 primer; C: ISSR835 primer; D: ISSR873 primer. Lane M represent DNA Mark (TaKaRa, Dalian, China). Lanes 1-7 represent the original parents Min43, Min62, Min56, Min54, Min53, Min50, Min49, respectively. Lanes 8-12 represent F2 parents 27-5, 41-4, 46-2, 51-2, 56-2, respectively. Lanes 13-31 represent diallel crosses of F2 generation C1-C19, respectively.

Table S1. Statistics of the *C. oleifera* F2 population's seedling traits.

Traits	Min value	Max value	Range	Mean±SD	Skewness	Kurtosis	CV %
H_S (cm)	10.25	17.77	7.52	13.14±2.04	1.18	1.16	15.53
D_S (cm)	2.08	3.05	0.97	2.49±0.26	0.86	0.35	10.63
H_S/D_S	4.13	7.53	3.40	5.32±0.95	1.11	0.85	17.85
Tr ($mmol \cdot m^{-2} \cdot s^{-1}$)	0.23	0.66	0.43	0.41±0.11	0.40	0.02	26.99
Gs ($\mu mol \cdot m^{-2} \cdot s^{-1}$)	14.20	40.35	26.15	24.86±6.82	0.40	-0.01	27.43
Pn ($\mu mol \cdot m^{-2} \cdot s^{-1}$)	1.49	5.06	3.57	2.57±0.90	1.23	1.90	34.88
Ci (ppm)	65.94	299.17	233.23	201.39±68.11	-0.21	-0.88	33.82
WUE	2.98	11.70	8.72	6.82±2.59	0.11	-1.03	37.95

SD: standard deviation; CV: coefficient of variation.H_S: seedling height; D_S: seedling ground diameter; H_S/D_S: height/diameter ratio; Tr: transpiration rate; Gs: stomatal conductance; Pn: photosynthetic rate; Ci: intercellular CO_2 concentration; WUE: water use efficiency.

Table S2. Fruit traits statistics of the 19 F2 families.

Traits	Min value	Max value	Range	Mean±SD	Skewness	Kurtosis	CV %
H_T (m)	1.83	2.75	0.92	2.22±0.24	0.36	0.09	10.66
D_T (cm)	5.5	8.8	3.3	6.74±0.98	0.57	-0.65	14.59
CA (m^2)	2.85	5.66	2.81	4.43±0.8	-0.25	-0.91	18.01
FH (mm)	20.61	40.35	19.74	31.1±4.22	-0.82	2.87	13.57
FD (mm)	21.75	37	15.25	29.56±3.32	-0.15	1.30	11.26
FSI	0.82	1.18	0.36	1.05±0.1	-1.07	0.17	9.65
PT (mm)	2.48	5.12	2.64	3.98±0.74	-0.58	-0.08	18.69
NSF	1.69	10.14	8.45	5.87±2.48	0.24	-0.90	42.23
FFW (g)	9.9	36.93	27.03	21.00±5.83	0.69	2.35	27.77
FPW (g)	3.68	20.22	16.54	11.65±3.81	-0.24	1.18	32.74
FSW (g)	6.22	16.72	10.5	9.35±2.84	1.37	1.04	30.41
FWK (g)	3.92	10.77	6.85	6.14±1.77	1.21	1.43	28.83
DWF (g)	6.28	22.17	15.89	12.37±4.19	0.82	0.19	33.89
DWP (g)	2.33	12.13	9.8	6.76±2.27	-0.08	1.22	33.54
DWS (g)	3.18	10.46	7.28	5.61±2.34	1.25	0.02	41.80
DWK (g)	1.36	5.85	4.49	3.20±1.28	0.72	-0.39	40.00
WCF (%)	0.23	0.61	0.38	0.41±0.1	-0.16	-0.16	23.06
KMC (%)	0.33	0.72	0.39	0.48±0.12	0.15	-1.15	25.21
FSR (%)	35.47	64.72	29.25	45.34±9.05	0.94	-0.12	19.96
YPP (kg)	4.75	15.58	10.05	8.82±3.14	-0.40	0.91	35.93

SD, standard deviation; CV, coefficient of variation; H_T, height of the tree; D_T, ground diameter of the tree; CA, crown area of the tree; FH, fruit height; FD, fruit diameter; FSI, fruit shape index; PT, pericarp thickness; NSF, number of seeds per fruit; FFW, single fruit fresh weight; FPW, fresh pericarp weight; FSW, fresh seed weight; FWK, fresh weight of kernel; DWF, dry weight of fruit; DWP, dry weight of pericarp; DWS, dry weight of seed; DWK, dry weight of kernel; WCF, water content of fruit; KMC,kernel moisture content; FSR, fresh seed rate; YPP, yield per plant.

Table S3. Significantly associated **10** ISSR loci of the 19 families in *C. oleifolia* F2 generation.

Family ID	835-3	835-12	844-9	858-2	858-3	858-10	827-3	827-5	820-7	820-8	873-4	815-3	815-5	815-6	815-11	815-12	845-7	845-9	845-11
C1	-	-	-	-	-	-	√♂	-	-	-	√	-	-	-	-	-	-	√♀	-
C2	√♂♀	-	√	√♀	-	-	-	-	-	-	√	-	-	√♂♀	-	-	-	-	-
C3	-	-	-	-	√♂	-	-	-	-	-	√	-	-	-	-	-	-	√♀	-
C4	√♀	-	-	-	-	-	-	-	-	-	-	-	-	-	-	-	-	-	√♂
C5	-	-	-	-	-	-	√♂	-	-	-	-	-	-	-	-	-	-	-	√♂♀
C6	√♂	-	-	-	-	-	-	-	-	-	-	-	-	-	-	-	√	-	√♀
C7	√♂	-	-	-	-	-	-	-	-	-	-	-	-	-	-	-	√	-	√♀
C8	√	-	-	-	√♂	√	-	-	-	-	-	-	-	-	-	-	√	-	√♀
C9	√♀	√♀	-	-	-	-	√♂	-	-	-	-	-	-	-	√	-	-	-	√♂
C10	√♂♀	√♀	-	-	-	-	-	-	-	-	-	-	-	-	√	-	-	-	√
C11	-	√♂♀	-	-	-	-	-	-	-	-	-	-	-	-	√	-	-	-	√♂
C12	-	√♀	-	-	√♂	-	-	-	-	-	-	-	-	-	√	-	-	-	-
C13	-	-	√	√	√♀	-	√	√♀	√♀	-	-	-	√♀	-	√	-	-	-	√♀
C14	-	-	-	-	√♀	-	√	-	√♀	-	-	-	-	-	√	-	-	-	√♀
C15	-	-	-	-	√♂♀	-	√	-	-	-	-	-	√♀	-	√	-	-	-	√♀
C16	-	√♂	-	-	-	-	√	-	-	-	-	-	-	-	√	-	-	-	-
C17	-	-	-	-	-	-	-	-	-	-	-	-	-	-	√	√	-	-	-
C18	-	√♂	√	-	-	-	-	-	-	-	-	-	-	-	√	√	-	-	-
C19	-	√	-	-	-	-	-	-	-	-	-	-	-	-	√	√	-	-	-
PRF	0.57	0.57	0.00	0.50	0.67	0.00	0.00	1.00	1.00	-	0.00	-	1.00	1.00	0.00	0.00	0.00	1.00	0.64
PRM	0.57	0.43	0.00	0.00	0.50	0.00	0.43	0	0.00	-	0.00	-	0.00	1.00	0.00	0.00	0.00	0.00	0.27

PRF, polymorphism ratio of partial female; PRM, polymorphism ratio of partial male.

Table S4. ISSR primers information.

Primer No.	*Primer sequence reverse primers (5'to 3')*
815	CTC TCT CTC CTC CTC TG
820	GTG TGT GTG TGT GTG TG
821	GTG TGT GTG TGT GTG TT
825	ACA CAC ACA CAC ACA CT
827	ACA CAC ACA CAC ACA CG
835	AgA gAg AgA gAg AgA gYC
844	CTC TCT CTC TCT CTC TRC
845	CTC TCT CTC TCT CTC TRg
856	ACA CAC ACA CAC ACA CYA
858	TGT GTG TGT GTG TGT GRT
865	ATg ATg ATg ATg ATg Atg
873	gAC AgA CAg ACA gAC A

Table S5. ISSR-PCR reaction system.

Component	Company	Initial concentration	Volume (μl)
Mg^{2+} (10 mmol·L^{-1})	Takara	2 mmol·L^{-1}	4
Primer (10 μmol·L^{-1})	Sangon Biotech	0.8 μmol·L^{-1}	1.6
dNTP (2.5 mmol·L^{-1})	Takara	0.3 mmol·L^{-1}	2.4
DNA (20 ng·μl^{-1})	Takara	30 ng·μl^{-1}	1.5
Taq enzyme (5 U·μl^{-1})	Takara	2.5 U·μl^{-1}	0.5
ddH_2O	-	-	10
